# 原发性肺非霍奇金淋巴瘤1例并文献复习

**DOI:** 10.3779/j.issn.1009-3419.2011.06.16

**Published:** 2011-06-20

**Authors:** 勇 梁, 蓉 付, 珊 高, 喜凤 董, 宗鸿 邵

**Affiliations:** 300052 天津，天津医科大学总医院血液科 Department of Hematology, Tianjin Medical University General Hospital, Tianjin 300052, China

**Keywords:** 肺肿瘤, 诊断, 治疗, 病理, Lung neoplasms, Diagnosis, Terapy, Pathology

## Abstract

原发性肺非霍奇金淋巴瘤（primary pulmonary non-Hodgkin’s lymphoma, PPNHL）是指起源于肺内淋巴组织的恶性淋巴瘤，是结外淋巴瘤的一种罕见类型，本文通过报道1例老年女性患者PPNHL，并结合相关文献复习，探讨了PPNHL的临床特点、诊断、治疗方法，以提高诊断率。

## Abstract (en)

Primary pulmonary non-Hodgkin's lymphoma (PPNHL) is a kind of malignant lymphoma originated from lymphoid tissue of the lung. PPNHL is a rare type in extra nodal lymphoma. In this article, we reported one case of 57-year-old woman with PPNHL, whose clinical characteristics, radiological features, diagnosis and treatment was described and related literatures were reviewed.

原发性肺非霍奇金淋巴瘤（primary pulmonary non-Hodgkin’s lymphoma, PPNHL）是指起源于肺内淋巴组织的恶性淋巴瘤，是结外淋巴瘤的一种罕见类型，约占全部淋巴瘤的0.4%^[1]^。PPNHL在临床表现及影像学上缺乏特异性，常被误诊。PPNHL患者早期明确诊断后，对其实施相应的手术治疗或化疗，可以得到治愈或缓解^[2]^。因此，提高对PPNHL的认识对预后有重要意义。现将天津医科大学总医院血液科收治的1例被病理证实的PPNHL报道如下，并行相关文献复习。

## 1 病例资料

患者，女，57岁，主因“发热伴左胸痛4个月，加重1周”，于2007年12月5日第1次入院。患者入院前4个月无明显诱因出现发热，体温最高达40 ℃，就诊于当地医院，给予左氧氟沙星抗炎治疗后，体温逐渐下降，但出现左前胸隐痛，此后症状反复发作，伴咳嗽，无咯痰、无咯血，无心悸，无声嘶，体重下降约3 kg。入院前10天胸痛加重，在当地医院行CT检查发现“左上肺占位”。拟诊“左上肺癌”，为进一步诊治收入我院肺外科。患者既往38年前有颈部淋巴结核病史，已治愈。否认放射线及毒物接触史，无烟酒嗜好。入院后行CT检查（2007年12月7日）示左上肺叶软组织密度块影，考虑肿瘤性病变，纵膈受侵，建议穿刺活检（图 1A，图 1B）。支气管镜检查示左上叶前端开口粘膜见隆起，于该隆起处取活检及刷片。病理报告（2007年12月23日）示小细胞恶性肿瘤，免疫组化LCA阳性，CgA阴性，Syn阴性。肺癌标志物均阴性。后两次行左上肺穿刺活检，病理报告（2007年12月28日及2008年1月3日）示弥漫一致性小细胞。免疫组化：CD20弥漫阳性，CD45RO散在阳性，LCA阳性，CD3阴性，CK阴性，CgA阴性，证实为B细胞性非霍奇金淋巴瘤（B-NHL）（图 2）。骨髓活检显示增生活跃，粒红比例增高，未见明显淋巴细胞增生浸润。骨髓涂片显示增生活跃，三系增生，大致正常骨髓象。同时外周血IgH及IgH/Bcl-2基因重排均为阳性。于2008年1月4日转入血液科继续治疗。在血液科期间行B超检查示全身表浅淋巴结均无肿大，肝、胆、脾、双肾未见占位病变，腹膜后及腹主动脉旁未见肿块，LDH为600.0 U/L（正常参考值范围为94.0 U/L-250.0 U/L）。复查CT（2008年1月7日）示左上肺肿块基底与纵膈相连，考虑肿瘤同时累及纵膈、肺及左侧肺门，最后确诊为PPNHL。自2008年1月9日开始规律行8次R-CHOP方案化疗。具体方案为：利妥昔单抗（Rituximab，商品名：美罗华）600 mg，d1，VCR 2 mg，d1，THP 60 mg，d1，CTX 1.0 g d1，曲安西龙48 mg，d1-5。化疗间隔21天-28天。化疗后复查CT（2008年1月14日）与2008年1月7日CT比较，左上纵膈旁软组织肿块明显变小（图 1C）。同时复查LDH降至正常水平，外周血IgH及IgH/Bcl-2基因重排均转为阴性。患者胸痛症状好转，无发热、咳嗽等不适。8次R-CHOP方案化疗后，予干扰素300万单位皮下注射，每周3次维持治疗。现患者病情稳定，无不适主诉，门诊随诊。

**1:**
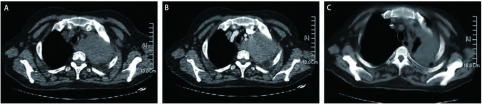
患者临床影像学特征。A：CT平扫：左上肺巨大肿块，侵犯纵膈；B：CT强化：肿块中度强化，包绕纵膈大血管，与食管分界不清；C：治疗后肿块明显缩小。 Clinical radiologic features of the patient. A: CT scan: a huge mass in the left upper lung invaded the mediastinum; B: CT contrast enhanced scan: mass medium sclerosis surrounded great vessels in the mediastinum, and no clear boundary with esophaged; C: the mass shrunk significantly after chemotherapy.

**2:**
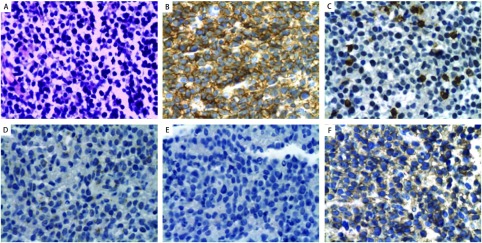
肺组织病理及免疫组织化学染色。A：弥漫一致性小细胞（HE, ×400）；B：CD20弥漫阳性（SP, ×400）；C：CD45RO散在阳性（SP, ×400）；D：CgA阴性（SP, ×400）；E：CK阴性（SP, ×400）；F：LCA阳性（SP, ×400）。 The pathology and immunohistochemical stains of lung tissue. A: diffused and consistent small cells (HE×400); B: diffused positive expression of CD20 (SP, ×400); C: scattered positive expression of CD45RO (SP, ×400); D: negative expression of CgA (SP, ×400); E: negative expression of CK (SP, ×400); F: positive expression of LCA (SP, ×400).

## 2 文献复习并讨论

肺脏是淋巴瘤最常侵及的器官，文献报道达25%-40% ^[1]^，但是原发于肺的淋巴瘤却非常少见，我科至今仅此1例。1993年Cordier等^[3]^对PPNHL提出如下诊断标准：①明确的病理组织学诊断依据；②病变局限于肺，可伴有或不伴有肺门、纵隔淋巴结受累；③确诊后3个月内无肺和支气管外组织或器官淋巴瘤。目前PPNHL的病理分期按Ferraro等^[1]^标准为依据。ⅠE期：仅累及肺或支气管（单侧或双侧）；Ⅱ1E期：累及肺和肺门淋巴结；Ⅱ2E期：累及肺和纵隔淋巴结；Ⅱ2E W期：累及肺和邻近的胸壁或膈肌；Ⅲ期：累及肺和胸廓处的淋巴结；Ⅳ期：广泛累及肺和其他组织或器官。本文报道的病例均符合该诊断标准，故诊断无疑，为Ⅱ2E W期。

PPNHL因临床症状和影像学表现缺乏特异性，很难与呼吸道其它疾病相鉴别，临床极易误诊。分析误诊原因：①淋巴瘤肺内浸润极为少见，临床医师对该疾病认识不足，思路狭窄，只考虑本专业范围内的疾病，而忽略了少见病^[4]^；②早期约半数患者缺乏症状，只是常规X线检查时被发现^[5]^，所以临床诊断十分困难。常见临床表现有咳嗽、胸痛、胸闷、盗汗、纳差、发热、体重减轻等，易误诊为肺炎、肺结核等常见病，本例患者发病时均有不同程度的呼吸道症状，如发热、胸痛等，但没有特异性，故而病情反复发作，一直按炎症行抗炎治疗，但疗效不佳；③肺淋巴瘤的影像学表现多种多样，病灶可以是单个，亦可为多个，肿块位于中心或边缘，病灶密度变化各异，给明确诊断造成困难。肺内淋巴瘤可分为四型^[6]^：（1）肿块、结节型：单发或多发，肿块呈类圆形，边界多较清晰，部分可形成空洞。此型易与肺癌相混淆。本文患者即属于此型，X线表现与原发性肺癌非常相似，起初被误诊为肺癌收入外科拟行手术治疗。最终通过肺穿刺病理检查才确诊为PPNHL；（2）炎症、肺泡型：淋巴瘤侵犯肺间质，并逐渐侵犯肺实质，表现为沿肺血管、支气管的斑片状密度增高影，边界模糊。肺炎在临床上是一种常见病，但当正规抗炎治疗无效时，我们需考虑淋巴瘤的可能；（3）支气管淋巴瘤型：少见。主要是淋巴瘤侵犯支气管内膜，表现为支气管管腔狭窄、闭塞，肺段、肺叶的阻塞性炎症或不张^[7]^；（4）粟粒性血液播散型：由于肺内淋巴瘤缺乏临床和影像学特征，诊断需要病理检查，有时多次活检亦不能确诊。对于此类患者应及时开胸或其它途径肺活检，以尽早明确诊断。本例即是通过两次CT引导下经皮肺肿块穿刺活检才得到诊断，有部分病人还需开胸肺活检才能明确诊断，提示该病诊断上的困难性。

PPNHL的临床预后取决于组织学类型和分期，该病一般病程长，进展缓慢，大部分是低度恶性，1年、5年和10年生存率在粘膜相关淋巴样组织（mucosa-associated lymphoid tissue, MALT）淋巴瘤患者中为91%、68%和53%，非MALT淋巴瘤患者中为85%、65%和64% ^[1]^。PPNHL中最常见的组织学亚型为低度恶性的MALT淋巴瘤，其次为弥漫大B细胞淋巴瘤，其它类型的淋巴瘤较为罕见。

PPNHL可选择的治疗方案包括手术、化疗或术后合并化疗。手术切除是无转移PPNHL首选治疗方法，术后病理若提示淋巴瘤，可辅以化疔。手术具有双重作用，即明确诊断和尽量切除病变组织。因为淋巴瘤的术后复发率可高达50%以上，故有建议手术后辅之以化疗。恶性淋巴瘤对化疗非常敏感，原发于肺的淋巴瘤也不例外，传统化疗常用CHOP或COP方案，针对CD20（+）的患者，应用Rituximab已成为NHL治疗的一条新途径。国内外较少采用放疗，主要考虑放疗后出现放射性肺损伤^[1, 8]^。本组NHL患者接受连续8个疗程的R-CHOP方案化疗后取得了良好效果，包块明显缩小，临床症状消失，现已存活3年半。但目前尚未见到有关PPNHL疗效的大宗病例报道。

尽管PPNHL表现很不典型，但临床表现和影像学资料能为诊断提供一些线索。尤其是当肺内病变不能用一般疾病解释或常规治疗效果不好时，应考虑到该病的可能性，并施以必要的有创检查，结合病理方法尽早确诊该病。目前，支气管纤维镜活检、CT引导下经皮肺肿块穿刺活检和VATS技术被公认为安全高、创伤小的检查方法，能提高早期诊断率，减少误诊^[9]^。
